# Sjögren's Syndrome: Concerted Triggering of Sicca Conditions

**DOI:** 10.1155/2019/2075803

**Published:** 2019-01-06

**Authors:** Zygmunt Mackiewicz, Justyna Mażul, Ieva Narkevičiūtė, Irena Dumalakienė, Irena Butrimienė, Rita Vilienė, Indrė Stankevičienė, Diana Mieliauskaitė

**Affiliations:** ^1^Department of Regenerative Medicine, State Research Institute Centre for Innovative Medicine, Santariskiu St. 5, LT-08406 Vilnius, Lithuania; ^2^Department of Immunology, State Research Institute Centre for Innovative Medicine, Santariskiu St. 5, LT-08406 Vilnius, Lithuania; ^3^Department of Chemistry and Bioengineering, Faculty of Fundamental Sciences, Vilnius Gediminas Technical University, Saulėtekio al. 11, LT-10223 Vilnius, Lithuania; ^4^Department of Experimental, Clinical and Preventive Medicine, State Research Institute Centre for Innovative Medicine, Santariskiu St. 5, LT-08406 Vilnius, Lithuania; ^5^Center of Rheumatology, Vilnius University, Santariskiu St. 2, LT-08406 Vilnius, Lithuania; ^6^Vilnius University Hospital Žalgiris Clinics, Lithuania

## Abstract

**Aim:**

The aim of this study was to evaluate the expression of persistence of mumps virus and some cells that interact with viral infection in the focus of the autoimmune epithelitis and peripheral blood of Sjögren's syndrome patients in comparison to patients with rheumatoid arthritis (RA) and nonautoimmune sicca syndrome (nSS).

**Materials and Methods:**

126 patients (119 women and 7 men) were grouped into four groups: (1) patients with primary Sjögren's syndrome (pSS), (2) patients with secondary Sjögren's syndrome due to rheumatoid arthritis (sSS), (3) patients with rheumatoid arthritis (RA), and (4) patients with nonautoimmune sicca syndrome (nSS). Immunohistochemical analysis of immune response to the suggested silent persistence of mumps virus in the minor labial salivary gland biopsies and flow cytometric analysis of blood cells was done.

**Results:**

Immunohistochemical signs of mumps virus persistence were found in the minor salivary glands of all study groups. Also, a significantly different immune response to virus infection (protein IFI16, interferons gamma and beta, dendritic cells, and receptor for natural killers) was revealed in the minor salivary glands of the study groups. Cytometric analysis of the blood cells revealed a dropping amount of circulating natural killers and dendritic cells in patients with SS. Significant correlations between immunohistochemical staining and serological findings were revealed.

**Conclusions:**

Abundant immunohistochemical signs of mumps virus protein in the salivary glands and depletion of circulating immune cells make a background for thought of presumable mumps or/and other virus participation in epithelial damage causing sicca syndrome in predisposed patients.

## 1. Introduction

Sjögren's syndrome is a common autoimmune disease characterised by sicca symptoms and extraglandular features. Etiology and detailed pathogenesis of Sjögren's syndrome (SS) are obscure [1]. Both innate and adaptive immune responses are implicated in the causation of SS, possibly triggered by viral infections and hormonal factors in a genetically susceptible host [2, 3].

The environmental triggers are believed to be infectious agents, which are most likely a virus [4, 5].

At the moment, conclusive evidence for a viral infection and the identity of such a virus remains elusive. Also, the wide spectrum of glandular and extraglandular manifestations in Sjögren's syndrome raises a hypothesis about the cooperation of infections agents in the mechanisms of this disease. Our earlier observations revealed a high frequency of mumps history in patients with primary and secondary Sjögren's syndrome, and the persistence of the mumps virus was documented by PCR in their saliva and minor salivary gland tissues (the data were presented at the international conferences). Mumps is caused by the mumps virus (MuV), a member of the *Paramyxoviridae* family of enveloped, nonsegmented, and negative-sense RNA viruses. Approximately one-third to one-half of MuV infections are asymptomatic or result in only mild respiratory symptoms, sometimes accompanied by fever [6].

The participation of external factors can indicate the elevated levels of *γ*-globulin, autoantibodies to nonspecific antigens such as rheumatoid factor, nuclear proteins, and cellular antigens SS-A/Ro, and SS-B/La [3]. Mumps virus enhancing hemagglutinin-neuraminidase activity and accelerating attachment to the host cells' lipid membrane is supposed to play such a role [6]. Response to infection by the production of autoantibodies often preludes clinical symptoms of autoimmunity [7]. There are reports that the La/SSB antigen involved in the processing of viral RNA has been found in some Sjögren's syndrome patients [8, 9].

Recent studies have discovered a substantial role of the activation of the type I interferon (IFN) in the pathogenesis of SS. Expression of IFNs in glandular tissues could support the idea of possible viral involvement in sicca syndrome pathogenesis [10, 11].

Dendritic cells (DC) and natural killers (NK) are the cells that first fight the virus invasion directly and produce IFNs impeding viral replication.

Therefore, our goal is to evaluate the expression of the persistence of mumps virus and some cells that interact with viral infection in the focus of the autoimmune epithelitis and peripheral blood of Sjögren's syndrome patients in comparison to patients with rheumatoid arthritis (RA) and nonautoimmune sicca syndrome (nSS).

## 2. Patients and Methods

### 2.1. Patients

Patients enrolled in the study were recruited from the Rheumatology Center of Vilnius University Hospital Santara Clinics. 126 patients (119 women and 7 men) were grouped into four: (1) patients with primary Sjögren's syndrome (pSS), (2) patients with secondary Sjögren's syndrome due to rheumatoid arthritis (sSS), (3) patients with rheumatoid arthritis (RA), and (4) patients with nonautoimmune sicca syndrome (nSS). The diagnosis of pSS, sSS due to RA, and nSS was firstly established before enrolling in this study. The diagnosis of pSS and sSS was based on the inclusion and exclusion criteria as defined in the American-European Consensus Group criteria for Sjögren's syndrome [12]. RA was diagnosed according to the 2010 ACR/EULAR classification criteria for rheumatoid arthritis [13, 14]. Patients in the nonautoimmune sicca syndrome group did not fulfill the SS nor RA classification criteria. All participants of the study underwent an extensive medical examination, whole blood analysis, and serologic evaluations (Table 1). All study patients were born before 1967, prior to the introduction of the mumps-measles-rubella vaccine. No patients were on immunosuppressive medications during this study. All subjects signed informed consent. The permission for research was gained from the Vilnius Regional Biomedical Research Ethics Committee (2014-05-20, no: 158200-14-733-248).

### 2.2. Histological and Immunohistochemical Analysis

Minor labial salivary glands (LMSGs) were collected for differential diagnosis by the oral specialist. Five or six minor salivary gland lobules were harvested and placed into a formalin fixative. Standard paraffin preparations were prepared, sectioned, and stained with hematoxylin and eosin for histopathological evaluation, with a picrosirius solution for the estimation of fibrosis and for immunoperoxidase immunohistochemical analysis (IHC). For IHC, 7 samples from each patient group were selected randomly.

The slides were examined for the presence of lymphocytic infiltrates by two board-certified pathologists.

For IHC analysis, the following primary antibodies were used: mouse monoclonal against mumps virus nucleoprotein (Thermo Fisher Scientific, clone 7B10, lot 1222, dilution 1 : 300), mouse monoclonal against human IFN-*β* (Santa Cruz, sc-73302, dilution 1 : 50), rabbit polyclonal against human IFN-*γ* (Abcam ab9657, dilution 1 : 200), mouse monoclonal against human IFI16 (Abcam ab55328, dilution 1 : 50), rabbit polyclonal against human NKG2D (Abcam ab203353, dilution 1 : 500), and rabbit monoclonal against human CD11c (Abcam ab52632, dilution 1 : 200).

Slides were deparaffinized in xylene, followed by rehydration in descending ethanol concentrations, from absolute to water. Endogenous peroxidase was quenched in 0.3% hydrogen peroxide for 5 min at room temperature (RT). Antigens were retrieved using citrate buffer pH 6.0 in a microwave histoprocessor *Milestone* for 20 min at 98°C. Then, sections were cooled down for 30 min at RT and incubated with primary antibody in dark humidity chamber at 4°C overnight, followed by incubation with secondary antibody from Dako detection kit (Dako REAL™ EnVision™ Detection System, Peroxidase/DAB+, Rabbit/Mouse) for 30 min at RT and Dako 3,3′-diaminobenzidine staining. Finally, the sections were counterstained with Mayer hematoxylin and embedded in glycergel mounting medium. All protocol procedure steps were followed by washing with PBS. For negative staining control, primary antibodies were replaced with normal serum of animal in which a secondary antibody was raised in the same dilution as the primary antibody or with antibody diluent. Staining results were evaluated independently by two board-certified pathologists and scored by a simple score value scheme using a light microscope *Olympus BX51* equipped with Nikon digital camera dxm1200.

Score values for stain intensity and stain outspread in a high-power microscopic magnification field (HPF) were shown as follows: *stain intensity*—0 = no specific staining, 1 = moderate specific staining, 2 = strong specific staining, and 3 = very strong specific staining—and *stain outspread*—0 = no specifically stained profiles, 1 = ≤10% of specifically stained profiles in HPF, 2 = ≤30% of specifically stained profiles, 3 = up to half of specifically stained profiles, 4 = ≤70% of specifically stained profiles, and 5 = up to 100% of specifically stained examined profiles in HPF.

### 2.3. Flow Cytometry Analysis

For assessment of cell surface phenotypic markers, peripheral blood from the study patients was stained with fluorescein isothiocyanate- (FITC-), phycoerythrin- (PE-), allophycocyanin- (APC-), and peridinin chlorophyll protein- (PerCP-) conjugated murine monoclonal antibodies (mAbs). For dendritic cell detection, Lin cocktail CD3/14/16/19/20/56-FITC, HLA-DR-PerCP, pDC marker CD123-PE, and cDC marker CD11c-APC (BioLegend, USA) were used; for NK, CD3-FITC and CD16+CD56-PE (Simultest™, Becton Dickinson, UK). Isotype controls were obtained from BioLegend. In test tubes containing 50 *μ*l of peripheral blood supplemented with anticoagulant, appropriate amounts of mAbs, according to the manufacturer's recommendations, were added. All samples with labeled antibodies were incubated for 30 min at RT in the dark. After incubation, red blood cells were lysed for 15 min with 160 mM NH_4_Cl solution at RT in the dark, and then the samples were centrifuged (500×*g*). Precipitated cells were washed with Cell Wash Buffer (Becton Dickinson, USA) and analyzed by two- or four-color flow cytometry on a FACSCalibur flow cytometer and CELLQuest software after calibration with CaliBRITE beads (BD Biosciences, San Jose, CA, USA). Data for each sample were acquired until 100,000 cells were analyzed.

### 2.4. Statistics

Statistical differences were analyzed using the Mann-Whitney *U* test (unpaired samples), and correlations were assessed by Spearman's rank test using the GraphPad Prism 6.0 software (GraphPad Software, San Diego, CA, USA). *P* values less than 0.05 were considered significant.

## 3. Results

### 3.1. Histology and Immunohistochemistry

Analysis of the salivary gland tissue showed histopathological heterogeneity amongst SS patients, including an extent of the cellular inflammatory infiltrate and the degree of epithelial damage in acini and ducts, healing either replacement of parenchymal glandular tissue by fat or fibrosis. pSS sialadenitis revealed a focal proliferation of ductal epithelial cells combined with focal lymphocytic infiltration and atrophy of acini. LMSG specimens were recognized as pSS using hematoxylin-eosin-stained sections when scored more than one focus of 50 or more lymphocytes per 4 mm^2^ of salivary tissue [12].

**Figure 1:**
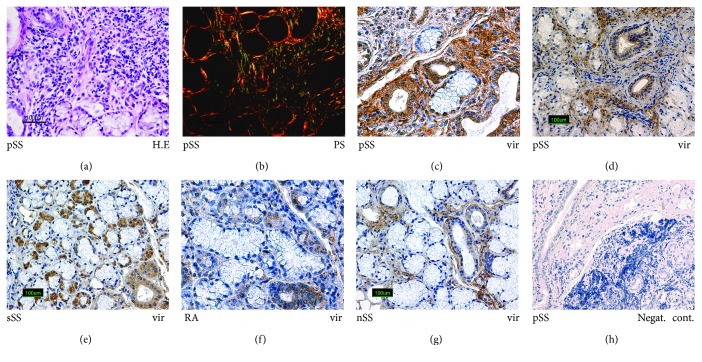
Expression of mumps virus protein in the minor salivary glands of patients with pSS, sSS, RA, and nSS. Histological and immunoperoxidase staining (IHC) in minor labial salivary glands: (a) H&E; (b) picrosirius-polarizing light; (c–g) mumps virus protein in the salivary glands of the study groups. Original magnification ×200. (h) Negative staining control.

The typical pSS pathological picture included lymphocytic infiltrations (Figure 1(a)) between acini and ducts, occasional infiltration of plasma cells and other inflammation-related mononuclear cells, and the formation of lymphoid follicle-like structures and fibrosis (Figure 1(b)). Histological picture characteristic for chronic sialadenitis was also found in sSS and RA. In nSS patients' LMSG biopsies dominated signs of parenchymal atrophy and fat deposition.

*Mumps virus protein* was found in the part of the ductal cells, in some acinar epithelial cells (Figure 1(c)), and in the parts of connective tissue closely surrounding acini and ducts in the biopsies of the pSS patients (Figure 1(d)). The foci of lymphocyte infiltration were mostly virus protein-free, with a little amount positively stained mononuclear cells in pSS patients. In the biopsies from sSS patients' the distribution and intensity of positive mumps virus protein staining profiles were similar to pSS (Figure 1(e)). In the biopsies from RA patients (Figure 1(f)), the number of positively mumps virus protein stained acini was much smaller in comparison to pSS and sSS. In nSS patients (Figure 1(g)) virus protein was found in some acinar cells, few mononuclear cells, and some parts of the stroma situated closely to acini and ducts.

**Figure 2:**
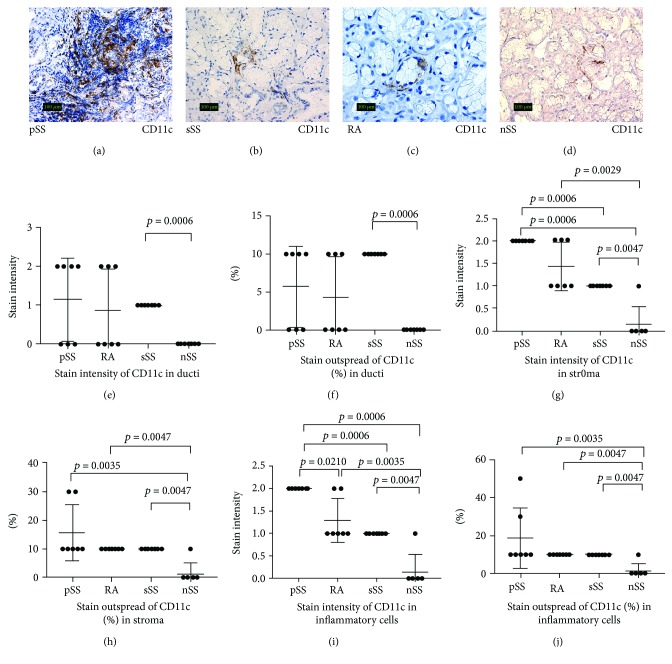
Expression of CD11c^+^ in the salivary glands of patients with pSS, sSS, RA, and nSS. (a–d) Histological staining (IHC) view of CD11c^+^ expression in the salivary glands of all study groups. Original magnification ×200. (e–j) Comparison of stain intensity and outspread of CD11c^+^ in the salivary glands of all study groups.

Our study revealed significant differences of the staining intensity and outspread of *CD11c antigen* in the inflammatory cells and stroma of minor salivary glands from pSS patients in comparison to sSS, RA, and nSS patients (Figures 2(a), 2(b), 2(c), 2(d), 2(g), 2(h), 2(i), and 2(j)). The stain intensity and outspread of *CD11c antigen* were similar in ducts from pSS, sSS, and RA patients, but comparison between sSS and nSS patients showed a significant difference in stain intensity and outspread in ducts (Figures 2(a), 2(e), and 2(f)).

**Figure 3:**
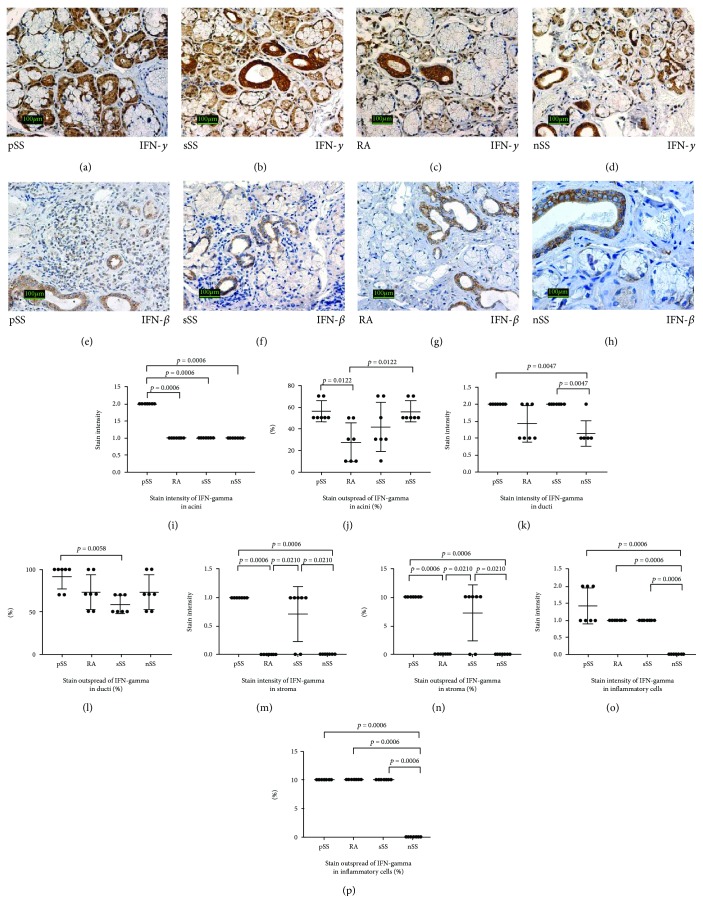
Expression of interferons beta and gamma in the salivary glands of patients with pSS, sSS, RA, and nSS. (a–h) Histological and immunoperoxidase staining (IHC) view of the expression of interferons beta and gamma in the salivary glands of all study groups. Original magnification ×200. (i–p) Comparison of stain intensity and outspread of interferons gamma in the salivary glands of all study groups.

The significantly highest stain intensity of *IFN-gamma* was found in acini of pSS patients (Figures 3(a), 3(d), and 3(i)), but stain outspread of IFN-gamma was similar to nSS (Figure 3(j)). The stain intensity of IFN-gamma in ducts showed significant differences between pSS and nSS patients and between sSS and nSS patients (Figure 3(k)). On the other hand, the significantly higher stain outspread of IFN gamma in ducts was found in pSS patients in comparison to sSS (Figure 3(l)). The stain intensity and outspread of IFN-gamma in the stroma of patients with pSS and sSS were similar and significantly higher in comparison to nSS and RA (Figures 3(a), 3(b), 3(c), 3(d), 3(m), and 3(n)). The significantly lowest stain intensity and outspread were revealed in the inflammatory cells of nSS patients (Figures 3(d), 3(o), and 3(p)).

Immunohistochemical staining of *IFN-beta* was weaker than IFN-gamma and similar in all study patients groups, but a little stronger in ducts than in acini of patients with pSS (Figure 3(e)), sSS (Figure 3(f)), RA (Figure 3(g)), and nSS (Figure 3(h)).

**Figure 4:**
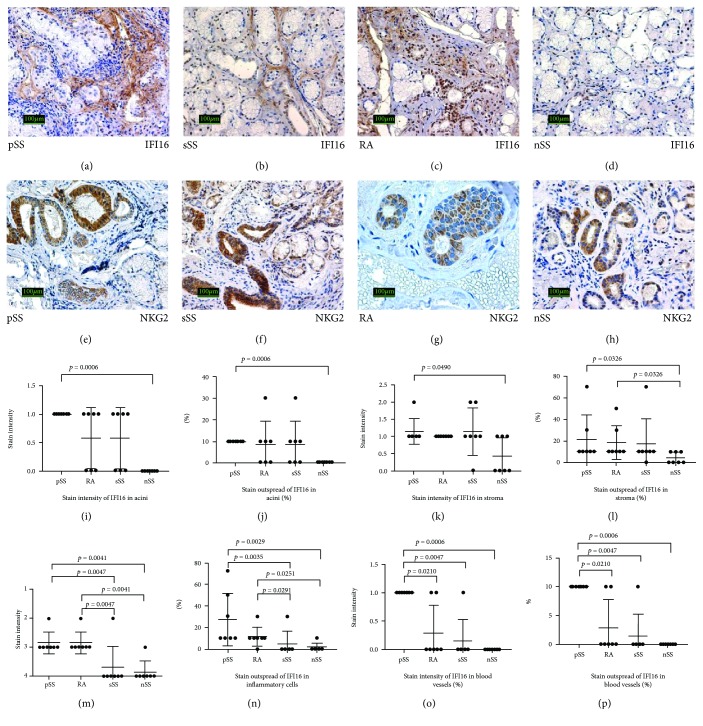
Expression of IFI16 and NKG2 in the salivary glands of patients with pSS, sSS, RA, and nSS. (a–h). Histological and immunoperoxidase staining (IHC) view of the expression of IFI16 and NKG2 in the salivary glands of all study groups. Original magnification ×200. (i–p) Comparison of stain intensity and outspread of IFI16 in the salivary glands of all study groups.

The analysis of stain intensity and outspread of *IFI16 protein* in acini and stroma revealed significant differences between pSS and nSS patients. Also, stain outspread of IFN16 in stroma showed a significant difference between RA and nSS patients (Figures 4(a), 4(b), 4(c), 4(d), 4(l), 4(j), 4(k), and 4(l)). The significantly different stain intensity and outspread of IFN16 protein in the inflammatory cells and blood vessels were found between all groups, except that stain intensity and outspread of IFN16 protein in blood vessels were similar in RA and sSS patients (Figures 4(m), 4(n), 4(o), and 4(p)).

*NKG2 receptor for natural killer* was detected as mosaic distribution in the cells of ducts (Figure 4) and in some inflammatory infiltrating cells in all study groups (Figures 4(e), 4(f), 4(g), and 4(h)).

#### 3.1.1. Analysis of DC and NK by Flow Cytometry

**Figure 5:**
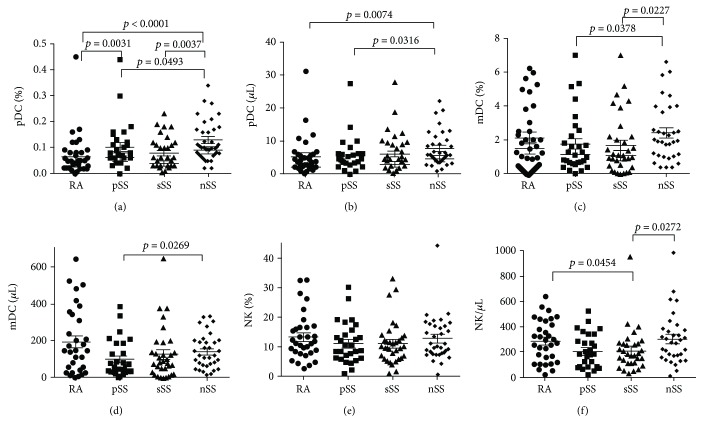
Expression of dendritic cells (DCs) and natural killers (NK) in the peripheral blood of pSS, sSS, RA, and nSS patients (flow cytometric analysis).

Distribution analysis of pDC (CD123^+^) showed that the highest frequency was observed in nonautoimmune sicca syndrome patients' group, and this frequency was significantly higher in comparison with RA (*p* < 0.0001), pSS (*p* = 0.0493), and sSS (*p* = 0.0037) groups (Figure 5(a)). Significant differences were found between RA and pSS groups. pDC absolute count was significantly lower in RA and pSS study group compared with nSS (accordingly, *p* = 0.0074 and *p* = 0.0316) (Figure 5(b)). Significant differences in the distribution of mDC (CD11c^+^) were found between pSS and nSS (*p* = 0.0378) and between sSS and nSS group (*p* = 0.0227) (Figure 5(c)). mDC absolute count was significantly lower in pSS study group compared with nSS (*p* = 0.0269) (Figure 5(d)).

The frequency of NK cells was similar in all study groups, and only absolute NK count was significantly reduced in sSS group compared with nSS and RA groups (accordingly, *p* = 0.0272, *p* = 0.0454) (Figures 5(e) and 5(f)).

Reliable positive correlation between immunohistochemical staining in the LMSG and serological findings was established. In the RA group, the frequency of pDC in blood samples strongly correlated with the stain intensity of IFN-*β* in ducts (*p* < 0.0001, *r* = 0.988) and stain intensity and stain outspread of NK cells (NKG2^+^) in LMSG (*p* < 0.0001, *r* = 0.988^∗^). More correlations were found in pSS group. In RA and in pSS groups, the frequency of pDC in blood samples strongly correlated with the stain intensity of IFN-*β* in the ducts (*p* = 0.022, *r* = 0.827), while mDC strongly correlated with the stain outspread of IFN-*γ* in the acini (*p* = 0.016, *r* = 0.748). Moreover, the frequency of NK cells in blood samples strongly correlated with the stain intensity and stain outspread of IFN-*β* in the acini (*p* = 0.011, *r* = 0.915^∗^) and also with the stain intensity and stain outspread of pDC in LMSG blood vessels (*p* = 0.045, *r* = 0.822^∗^) and stain intensity and stain outspread of mumps virus protein in ducts (accordingly, *p* = 0.045, *r* = 0.822 and *p* = 0.047, *r* = 0.818). In sSS group, only the frequency of NK cells in blood samples strongly correlated with the stain intensity of IFI16 in the ducts and stroma (accordingly, *p* = 0.003, *r* = 0.922 and *p* = 0.001, *r* = 0.945) and stain intensity and stain outspread of mumps virus protein in LMSG acini (*p* = 0.003, *r* = 0.922^∗^). In nSS group, a strong correlation was established between the frequency of mDC cells in the blood samples and stain intensity of IFN-*β* in LMSG ducts (*p* < 0.002, *r* = 0.939), as well as with stain intensity of IFN-*γ* in LMSG ducts (*p* < 0.028, *r* = 0.806).

^∗^The score values of stain intensity and stain outspread are the same.

## 4. Discussion

Both innate and adaptive immune responses are implicated in the causation of SS, possibly triggered by viral infections and hormonal factors in a genetically susceptible host [2, 3]. At the moment, conclusive evidence for a viral infection and the identity of such a virus remains elusive. The wide spectrum of glandular and extraglandular manifestations in Sjögren's syndrome raises a hypothesis about the cooperation of infections agents in the mechanisms of this disease. According to our earlier observations about mumps, we set tasks to evaluate the persistence of mumps virus in the salivary glands. In our study mumps virus protein was found in the minor salivary glands of pSS, sSS, RA, and nSS patients. The distribution and intensity of positive mumps virus protein in minor salivary glands were similar in pSS and sSS patients. In minor salivary glands of RA patients, the number of positively mumps virus protein stained acini was much smaller in comparison to pSS and sSS. In nSS patients virus protein was found in some acinar cells and some parts of the stroma situated closely to acini and ducts. These findings support an idea that mumps virus participate in epithelial damage causing sicca syndrome.

Also, according to recent data, salivary gland epithelial cells (SGEC), once believed to be passive onlookers, seem to be the nidus of pathogenetic events in SS. SGEC in SS, therefore, are active players in the inflammatory and autoimmune response. Once activated, these cells orchestrate innate and adaptive immune response [15].

If Sjögren's syndrome is caused by a virus, there should be a persistent latent infection when the viral pathogen after acute infection remains present in the infected host causing sporadic reactivation events. Expression of CD11c, IFN-gamma, IFN-beta, and NKG2 receptor for natural killer in the salivary glands of our study groups demonstrated these events.

Significant differences of the staining intensity and outspread of CD11c antigen in the inflammatory cells and stroma of minor salivary glands from pSS patients in comparison to sSS, RA, and nSS patients and ducts from sSS in comparison to nSS demonstrate an activity of dendritic cells in minor salivary glands.

Both plasmacytoid dendritic cells (pDCs) and classic dendritic cells (cDCs) have a role in SS pathogenesis. Plasmacytoid DCs (pDCs) are present in the lymphocytic foci of salivary glands in patients with SS but not in healthy controls [15, 16]. Dendritic cells capture antigens and regulate reactions of adaptive immune responses [16]. Plasmacytoid DC producing CD123—the molecule consisting of three extracellular domains [17] selectively express endosomal TLR7 and TLR9 that detect virus nucleic acids. TLR7 or TLR9 triggers a downstream signaling cascade resulting in the secretion of IFN-*α*/*β* [18]. pDC—the professional IFN-*α*/*β*-producing cells were found to be reduced in the periphery and recruited preferentially in the minor salivary glands of SS patients [19]. Earlier our finding of a significant decrease of circulating pDC cells in RA and sSS due to RA patients and decrease of conventional mDC in SS patients supports the suggestion that DC populations may play a substantial role in autoimmune diseases and are exhausted in fighting viruses [11].

Principal cells that directly fight the viruses by means of IFNs are DC, cytotoxic T lymphocytes (CTL), NK, and natural killer T (NKT) cells [20]. The IFNs are a group of inflammatory cytokines that inhibit viral replication and regulate the immunity of host [21]. Like other paramyxoviruses, mumps virus initiates infection by attachment of the HN protein to sialic acid on the cell surface glycolipids and operates together with the F protein facilitating fusion with the plasma membrane [22]. IFNs disturb this process and in some cases are responsible for the initiation and perpetuation of the autoimmune disease [23–26]. Induction of IFN-*α*/*β* is based on the interactions between viral pathogen-associated molecular patterns (PAMPs) and host pattern-recognition receptors (PRRs) [27].

Though type I IFNs (IFN-*α*/*β*) were sought to be the predominant players in the pathogenesis of SS, recent data suggest an important role also of type II IFN (IFN-*γ*) in the immune sicca pathogenesis [10, 28]. IFN-*γ* is predominantly produced by T and NK cells to a lesser extent by dendritic cells, macrophages, and B cells [29]. IFN-*γ* promotes antimicrobial protection, apoptosis, inflammation, and also host tissue damage producing autoantigens. Both type I and type II IFNs have been thought to be implicated in the pathogenesis of Sjögren's syndrome [23].

The significantly highest stain intensity of IFN-gamma was found in the acini of pSS patients. The stain intensity of IFN-gamma in ducts showed significant differences between pSS and nSS patients and between sSS and nSS patients. The significantly higher stain outspread of IFN gamma in ducts was found in pSS patients in comparison to sSS. The significantly lowest stain intensity and outspread were revealed in the inflammatory cells of nSS patients.

Immunohistochemical staining of IFN-beta was weaker than IFN-gamma and similar in all study patients groups, but a little stronger in ducts than in acini. In accordance, recent research findings highlight a consistent ex vivo inhibitory effect of IFN-*β* on proinflammatory cytokine production and NO pathway in pSS patients. These data suggest that IFN-*β* could represent a potential candidate for targeting inflammation during pSS [30].

It is known that normal minor salivary gland cells do not constitutively express IFI16. Usually IFI16 expression is limited to the cell nuclei of hematopoietic cells, vascular endothelial cells, and keratinocytes [31]. It is also known that extracellular leakage of IFI16 breaks the tolerance to self-proteins and leads to the production of anti-IFI16 autoantibodies [32, 33]. Strong expression of the genes inducing IFNs could be a pivotal step in the development of primary Sjögren's syndrome. Our study revealed immunohistochemical evidence for the mislocation and extracellular leakage of IFI16 in the parenchymal salivary cells of patients of our study. We also found overexpressed and mislocated IFI16 in the lymphocytes of the minor salivary glands. We suppose that IFI16 may act as an infection-caused autoantigen involved in the development of autoimmunity. Also, such possibility has been postulated by other researchers [34].

NKG2 receptor for natural killer recognizing infected cell was detected as mosaic distribution in the cells of ducts and in some inflammatory infiltrating cells in all study groups.

Our study demonstrated the evidence of antiviral immune response according to the analysis of DC and NK in pSS, sSS, and RA patients' blood. Distribution analysis of pDC (CD123^+^) showed that the highest frequency was observed in nSS patients in comparison to RA, pSS, and sSS groups. pDC absolute count was significantly lower in RA and pSS study group compared with nSS. Significant differences in the distribution of mDC (CD11c^+^) were found between pSS and nSS and between sSS and nSS group. mDC absolute count was significantly lower in pSS study group compared with nSS.

The frequency of NK cells was similar in all study groups.

These serological findings and reliable positive correlations between immunohistochemical staining and serological findings could explain an idea that virus or viruses participate in epithelial damage causing sicca syndrome. Certainly, our study does not try to demonstrate that mumps virus is the only virus that is involved in the pathogenesis of SS. Probably mumps virus works on cooperation with another infection. One of the shortcomings of our research is that we analyzed small groups by immunohistochemistry.

Some authors suggest that rather than a specific infection, a response to self-antigens can initiate SS [35, 36].

Results of our study suggest that the pathogenesis of SS seems to be a multifactorial process leading to damage and dysfunction of the salivary glands. Possibly, environmental factors such as a viral infection affect the salivary glands and stimulate dendritic or/and glandular cells to activate the HLA-independent innate immune system, which using Toll-like receptors recognize pathogen-specific epitopes. This process leads to the upregulation of adhesion proteins and production of chemokines by the glandular epithelial cells [37].

Sjögren's syndrome is prevalent in patients of advanced age, especially in 50 years and older women [38]. Age-related disbalance in hormones, such as estrogens, prolactin, progesterone, and glucocorticoids, may affect DC differentiation, maturation, and function leading to a proinflammatory not to an anti-inflammatory or tolerogenic phenotype. These processes can trigger autoimmune disease [1, 39, 40].

The results of our study and analysis of data of other investigators suggest that even if the expression of a viral or virus-mimicry endogenous protein in the salivary gland are not sufficient to elicit a complete SS disease phenotype, some sicca pathogenesis presumably is a response to viral infection. Certainly, Sjögren's syndrome neither any autoimmune sicca condition is considered to be infectious or contagious diseases. Multiple factors, including genetic stability, unfavorable cellular tissue environment, and/or exposure to pathogens, are required to trigger a virus-mediated development of SS [32, 41]. Disbalanced microbiome also can disturb immune homeostasis and participate in SS triggering. Probably, this can explain that nonautoimmune sicca conditions, at least partially, share similar pathogenesis with autoimmune SS.

## 5. Conclusion

Abundant immunohistochemical signs of mumps virus protein in the salivary glands and depletion of circulating immune cells make a background for thought of presumable mumps or/and other virus participation in epithelial damage causing sicca syndrome in predisposed patients.

## Figures and Tables

**Table 1:** Clinical and serological characteristics of study patients.

Patient group features	pSS (*n* = 29)	RA (*n* = 32)	sSS (*n* = 32)	nSS (*n* = 33)
Age in years, mean (range)	57 (49–78)	54 (50–78)	59 (49–70)	59 (49–91)
Disease duration in years, mean ± SD	8 ± 5	10 ± 9	16 ± 10	8 ± 7
DAS28, mean ± SD	—	5.41 ± 1.43	5.59 ± 1.55	—
Duration of dryness in years, mean ± SD	6 ± 4	0	6 ± 5	6 ± 7
Schirmer's I test positive (≤5 mm/5 min), *n* (%)	26 (86.6)	0 (0)	23 (72)	6 (18)
Unstimulated salivary flow positive (≤1.5 mL/15 min), *n* (%)	25 (83.3)	0 (0)	13 (40.7)	2 (6)
Unstimulated salivary flow (mL/15 min), mean ± SD	1.36 ± 0.95	3.45 ± 1.36	1.80 ± 1.25	2.88 ± 1.44
Focus score positive (number of lymphocytic foci/4 mm^2^), *n* (%)	29 (100)	0	32 (100)	0 (0)
Positive autoantibodies				
RF, *n* (%)	14 (48.2)	20 (62.5)	23 (71.9)	—
ACCP, *n* (%)	1 (3.4)	24 (75)	16 (50)	—
ANA, *n* (%)	21 (72.4)	1 (3.1)	7 (21.8)	—
Anti-SSA^+^, *n* (%)	4 (13.8)	0 (0)	2 (2.25)	—
Anti-SSB^+^, *n* (%)	0 (0)	0 (0)–	0 (0)	—
Anti-SSA/SSB^+^, *n* (%)	18 (62.1)	0 (0)	3 (9.3)	—

## Data Availability

No data were used to support this study.
